# Genetic insight into the relationship between inflammatory bowel disease and *Clostridioides difficile* infection

**DOI:** 10.1128/msphere.00567-24

**Published:** 2024-10-22

**Authors:** Kelly C. Cushing-Damm, Yanhua Chen, Xiaomeng Du, Annapurna Kuppa, Chinmay Raut, Antonino Oliveri, Vincent L. Chen, Brett Vanderwerff, Matt Zawistowski, Krishna Rao, Peter Higgins, Elizabeth K. Speliotes

**Affiliations:** 1 Department of Internal Medicine, Division of Gastroenterology, University of Michigan, Ann Arbor, Michigan, USA; 2 Department of Internal Medicine, Division of Cardiology, University of Michigan, Ann Arbor, Michigan, USA; 3 Department of Biostatistics and Center for Statistical Genetics, University of Michigan, Ann Arbor, Michigan, USA; 4 Department of Internal Medicine, Division of Infectious Diseases, University of Michigan, Ann Arbor, Michigan, USA; 5 Department of Computational Medicine and Bioinformatics, University of Michigan, Ann Arbor, Michigan, USA; The University of Iowa, Iowa City, Iowa, USA

**Keywords:** *Clostridium difficile*, inflammatory bowel disease, genetics

## Abstract

Patients with inflammatory bowel disease (IBD) are at increased risk of *Clostridioides difficile* infection (CDI). Herein, we aimed to determine if genetic risk contributes to this observed association. We carried out a genome-wide association study (GWAS) analysis in the Michigan Genomics Initiative and the United Kingdom Biobank for CDI based on ICD codes and meta-analyzed these results with similar publicly accessible GWAS summary statistics from Finngen. Conditional and joint multi-SNP analyses were used to identify independent associations. Imputation of the human leukocyte antigen (HLA) region with fine mapping was used to try to identify causal HLA allele groups. Two-sample bidirectional Mendelian randomization (MR) was implemented to determine causal relationships between IBD and CDI. A total of 3,500 cases of CDI and 674,323 controls were meta-analyzed, revealing one genome-wide significant variant for CDI, *HLA-C;LINC02571*-rs3134745-C (*P* = 4.27E−08), which annotated to the major histocompatibility complex on chromosome 6. While fine mapping did not identify a statistically significant HLA allele group, there was a suggestive signal for HLA-B*35:01 (*P* = 4.74e−04). Using two-sample MR, genetically predicted IBD was associated with increased risk of CDI (MR Egger [odds ratio {OR} 1.16, 95% confidence interval {CI} 1.02–1.31]). Subset analysis revealed that risk was primarily driven by genetically predicted ulcerative colitis (MR Egger [OR 1.22, 95% CI 1.05–1.41]). These results highlight the importance of the host immune response in CDI pathogenesis, help explain the observed relationship between IBD and CDI, and open new avenues for targeted treatment of CDI in IBD.

**IMPORTANCE:**

Data from this paper (i) provide reproducible evidence that susceptibility CDI is genetically mediated, (ii) highlight genetic risk as a mechanism for the increased risk of CDI in patients with inflammatory bowel disease, and (iii) point toward anti-interleukin-23 therapy as a common therapeutic strategy.

## INTRODUCTION

*Clostridioides difficile* infection (CDI) occurs when the enteric bacteria, *C. difficile*, shifts to a toxigenic state, producing and releasing local exotoxins (1). These exotoxins mediate gastrointestinal injury, leading to symptoms such as watery diarrhea and abdominal cramping. A subset of patients may develop a more severe or fulminant form of the infection known as toxic megacolon, increasing the risk for perforation and sepsis. The public health burden of CDI infection is exceptionally high, with over 400,000 infections and over 20,000 in-hospital deaths estimated in the United States in 2017 (2).

Inflammatory bowel disease (IBD) is an established risk factor for both asymptomatic carriage of *C. difficile* and CDI. In a cohort of IBD patients in remission, *C. difficile* was found more often in the asymptomatic carrier state in the stool of IBD patients when compared to healthy controls (8.2% vs 1%, *P* = 0.02), suggesting that the altered intestinal landscape conferred by IBD is itself a risk factor for *C. difficile* colonization (3). IBD patients are also at increased risk of CDI in the outpatient (odds ratio [OR] 4.79, 95% confidence interval [CI] 3.79–5.80) (4) and inpatient setting (OR 2.9, 95% CI 2.1–4.1) (5). Unfortunately, CDI is associated with adverse outcomes in IBD including increased hospitalizations, longer hospital stays, escalation of medical therapy, higher rates of colectomy, and higher rates of mortality (6–8). Thus, there remains a strong need for continued investigation into the mechanisms that drive CDI, discovery of mitigatable risk factors, and identification of therapeutic targets.

Given the paucity of data investigating the impact of host genetics on the observed relationship between IBD and CDI, we aimed to determine if genetic variation influences the observed association between these two diseases.

## MATERIALS AND METHODS

### Genome-wide association study and meta-analysis

Details on data cohorts, genotyping, and imputation are included in the supplemental material. Genome-wide association study (GWAS) summary statistics for CDI in the FinnGen cohort were publicly available. Summary statistics, including variant chromosome and position, effect allele, other allele, beta effect size, standard deviation, effect allele frequency, and *P* value, were downloaded from the FinnGen website. Details of GWAS methodology can be found on the FinnGen website (https://finngen.gitbook.io/documentation/v/r5/data-download). Briefly, GWAS of autosomal variants was carried out using mixed modeling by Scalable and Accurate Implementation of Generalized mixed model (SAIGE, version 0.36.3.2), controlling for sex, age, principal components, and genotyping batch (9). GWAS summary statistics for CDI in the United Kingdom (UK Biobank [UKBB]) and Michigan Genomics Initiative (MGI) cohorts were computed by the study team. GWAS of autosomal variants was carried out for each cohort, using mixed modeling by SAIGE (version 0.29), with CDI as the dependent variable and single-nucleotide polymorphisms (SNPs) in an additive genetic model. The model controlled for the covariates sex, age, age2, and principal components 1–10. Only SNPs with an imputation quality cutoff of >0.85 were analyzed.

Meta-analysis of GWAS summary statistics for CDI was performed using the software METAL (release: 28 August 2018) (10). Input data included beta effect sizes and standard errors. The genomic control parameter was 0.892; therefore, no adjustment was made. The total number of variants after meta-analysis was 35,289,364. Genome-wide significance was defined as a *P* value of less than or equal to 5e-8. Given the relatively small cohort size, high-priority variants were identified using a *P* value (for association) of <1e−5, a *P* value for heterogeneity (pHet) of >0.05, a minor allele frequency (MAF) of >0.05, and a consistent direction of effect across all three cohorts. Conditional and joint multi-SNP analysis (COJO) was performed using GCTA software (version 1.91.2) (11) to distinguish independent loci. Approximate, stepwise conditional analyses were completed utilizing the full genotypes, including imputed genotypes, from the UK Biobank. Only variants with MAF of >0.01 were included in analyses. Linkage disequilibrium (LD) was assessed for variants within 10 MB, which is the default value for COJO. Details on association between genotypes and microbiome abundance are included in the supplemental data.

### Human leukocyte antigen imputation and fine mapping

Human leukocyte antigen (HLA) allele groups were imputed for individuals in the MGI and UKBB cohorts. The FinnGen cohort was excluded, given the lack of individual-level data. Imputation was completed on the Michigan Imputation Server using the hard-call genotype variants on chromosome 6, based on the four-digit multi-ethnic HLAv2 (GRCh37/hg19) reference panel (12, 13). The HLAv2 reference panel included 20,349 samples with 22,733 sites (570 HLA alleles, 3,449 HLA amino acids, 4,023 SNPs within HLA, and 14,691 scaffold SNPs) spanning chromosome 6, positions 27970031–33965553. Association analyses were carried out using an additive model in REGENIE, controlling for sex, age, age2, and principal components 1–10 (14). An imputation quality cutoff of >0.7 was used, and rare alleles (MAF < 0.01) were excluded from further analysis due to limitations in accuracy interpretation (15). Meta-analysis of summary statistics was performed using the inverse variance-based method (input: beta and standard error) in METAL. Genomic control correction was applied to each data set to account for population stratification or relatedness (UKBB: lambda = 1.843, MGI: lambda = 1.911). Association analysis was restricted to four-digit HLA allele groups, which were present in both cohorts. Significance was defined using a Bonferroni-corrected *P* value.

### Mendelian randomization

Details on bidirectional association of IBD and CDI risk variants are included in the supplemental data. For Mendelian randomization (MR) analyses, the *a priori* exposure of interest was IBD, and the outcome of interest was CDI. The instrumental variables (i.e., SNPs) for the exposure of interest and their associated summary statistics were extracted from a published meta-analysis of IBD susceptibility (16). Only instrumental variables which reached genome-wide significance (*P* < 5e−8) in the European cohort were included. Each instrumental variable was tested for bias using the *F*-statistic {*F*-statistic = [*r*^2^ × (*N* − 1 − *k*)]/[(1 − *r*^2^) × *k*]} (17). All instrumental variables with an *F*-statistic of <10 were excluded from analyses. Independent instrumental variables were identified using the clumping method in the TwoSampleMR package (version 0.5.6), which identifies the instrumental variable with the strongest association with the exposure of interest if multiple instrumental variables are located in the same region (18). The outcome data set included summary statistics generated from the meta-analysis of CDI described above. Two-sample MR was performed using the TwoSampleMR package in R (version 4.1.3) (19). MR Egger and inverse variance weighted (IVW) methods are reported. Both MR Egger and IVW measures were tested for heterogeneity. The Egger intercept was calculated to assess for directional pleiotropy, and sensitivity analysis was performed with MR-PRESSO. Scatter plots (SNP effect on exposure by SNP effect on outcome), forest plots (SNP effect size on outcome and leave one out analysis), and funnel plots were generated (Fig. S1 to S12). The statistical significance threshold was *P* < 0.05.

MR was also performed to assess for reverse causality, with CDI as the exposure of interest and IBD as the outcome of interest. All variants meeting an association *P* < 1e−5, a pHet of >0.05, a MAF of 0.05, and a consistent direction of effect across all three cohorts in the CDI meta-analysis were included in the exposure data set. The same methodologic approach was applied to identify independent SNPs. The outcome data set included 12,716,084 SNPs in 34,652 European individuals (“ieu-a-31”).^16^ Analyses were again performed using the TwoSampleMR package in R (19). MR egger and IVW significance values are reported along with tests for heterogeneity and directional pleiotropy. Scatter plots, forest plots, and funnel plots were generated and are provided in the supplemental data (Fig. S13 to S16).

### Approval

The UKBB analyses in this study were conducted under the UK BioBank Resource Project 18120. Details on data availability are included in the supplemental material.

## RESULTS

### Meta-analysis of CDI and fine mapping

**Fig 1:**
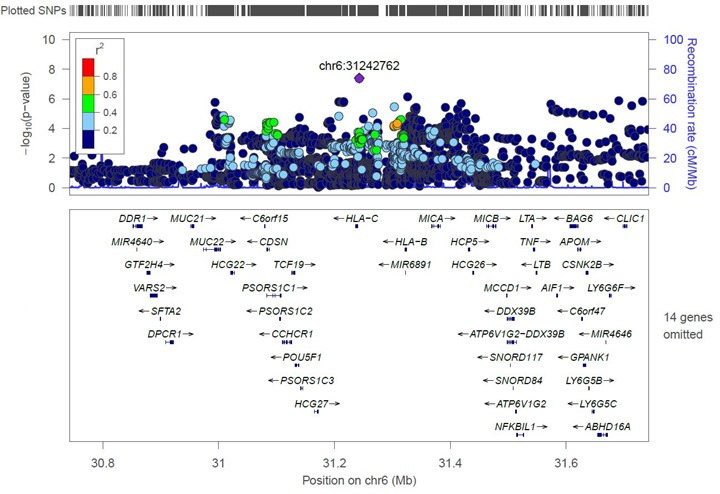
A LocusZoom plot demonstrating linkage disequilibrium between the genome-wide significant variant for CDI, rs3134745, and SNPs ± 500 kB of this variant.

GWAS summary statistics from three cohorts, including 3,500 cases of CDI (MGI [*n* = 1,229], UKBB [*n* = 830], and FinnGen [*n* = 1,441]) and 674,323 controls (MGI [*n* = 50,259], UKBB [*n* = 407,993], and FinnGen [*n* = 216,071]) were meta-analyzed (Table S2). One SNP, *HLA-C;LINC0257*-rs3134745-C (*P* = 3.91e−08), reached genome wide significance. After conditional and joint analyses (COJO), rs3134745-C remained genome-wide significant (β = 0.16, *P* = 4.27e−8) (Table 1). This variant annotated to an intergenic location between *HLA-C* and *LINC0257* in the major histocompatibility complex (MHC) on chromosome 6. A LocusZoom plot (±500 kB), using hg19/1000 Genomes Nov 2014 EUR as the reference, demonstrated LD across the region (Fig. 1). To identify a potentially causative HLA allele group in the region, fine mapping of chromosome 6 was performed in the MGI and UKBB cohorts. The rs3134745 variant was again identified as having the strongest association signal in this region (*P* = 5.18e−5). No HLA allele groups exceeded a Bonferroni-corrected statistical significance threshold of ≤2.54e−4 (0.05/197) (Table S3). However, a suggestive signal was observed for HLA-B*35:01 (*P* = 4.74e−4).

**TABLE 1:** Meta-analysis of genome-wide association study summary statistics identifies six independent genomic variants which are associated with *Clostridioides difficile* infection^*a*^

rsID	Chr	BP (build:hg19)	EA	OA	EA frequency	Beta	Standard error	*P* value	Function	Gene
rs3134745	6	31,242,762	t	c	0.328	−0.160	0.029	4.27E−08	Intergenic	*HLA-C;LINC02571*
rs10927954	1	14,245,266	c	g	0.097	0.185	0.038	8.72E−07	Intergenic	*PRDM2;KAZN-AS1*
rs12458428	18	71,331,397	a	c	0.328	0.124	0.027	3.23E−06	Intergenic	*LINC02582;FBXO15*
rs11707141	3	108,487,582	a	g	0.720	−0.124	0.027	5.22E−06	Intergenic	*RETNLB;TRAT1*
rs1182870	1	208,971,963	t	c	0.878	−0.179	0.039	5.98E−06	Intergenic	*LINC01717;LINC01774*
rs10031490	4	109,771,479	a	g	0.861	−0.171	0.038	8.54E−06	Intronic	*COL25A1*

^*a*^ BP, base position; Chr, chromosome; EA, effect allele; OA, other allele.

An additional 153 variants had an association *P* value of <1e−5 in the meta-analysis, with a corresponding p-Het of >0.05, a MAF of >0.05, and a consistent direction of effect across all three cohorts (Table S2). Of these 153 variants, 5 were found to be independently associated with CDI (at a *P* value of <1e−5) after COJO analysis (Table 1). These variants included *PRDM2;KAZN-AS1*-rs10927954-C (β = 0.19, *P* = 8.72e−7), *LINC02582;FBXO15*-rs12458428-A (β = 0.12, *P* = 3.23e−6), *RETNLB;TRAT1*-rs11707141-G (β = 0.12, *P* = 5.22e−6), *LINC01717;LINC01774*-rs1182870-C (β = 0.18, *P* = 5.98e−6]), and *COL25A1*-rs10031490-G (β = 0.17, *P* = 8.54e−6).

### MR

Genetically predicted IBD was tested for association with CDI. Instruments (i.e., SNPs) were extracted from the Liu et al. meta-analysis, which included combined summary statistics for GWAS and immunochip analyses of IBD susceptibility (16). There were 159 SNPs of which 106 were found to be independent after clumping. Of these, 105 were present in the outcome data set (CDI). Genetically predicted IBD was significantly associated with risk of CDI using both MR Egger (β = 0.15, *P* = 0.027; OR 1.16 [95% CI 1.02–1.31]) and IVW (β = 0.09, *P* = 0.001; OR 1.10 [95% CI 1.04–1.15]) methods (Table 2). There was no significant heterogeneity (MR Egger [*P* = 0.296] and IVW [*P* = 0.30]) and no directional pleiotropy (*P* = 0.37). Furthermore, the MR-PRESSO global test indicated no pleiotropy (*P* = 0.31).

**TABLE 2:** MR identifies significant effect of IBD susceptibility variants on CDI^*a*^^,^^*b*^

Exposure	Method	*N*	Beta	*P* value	OR
Outcome: CDI					
IBD	MR Egger	105	0.145	0.027	1.156 (1.018–1.312)
IBD	Inverse variance weighted	105	0.091	0.001	1.096 (1.040–1.154)
CD	MR Egger	97	0.082	0.188	1.085 (0.962–1.225)
CD	Inverse variance weighted	97	0.060	0.008	1.061 (1.016–1.109)
UC	MR Egger	62	0.197	0.010	1.218 (1.053–1.410)
UC	Inverse variance weighted	62	0.103	0.0003	1.109 (1.049–1.173)
Outcome: IBD					
CDI	MR Egger	5	0.178	0.669	1.195 (0.571–2.498)
CDI	Inverse variance weighted	5	0.054	0.424	1.055 (0.925–1.203)

^*a*^ Sensitivity analyses with disease subtypes highlight effect driven by UC susceptibility variants, rather than CD. *N* denotes the number of SNPs for the exposure variable.

^*b*^ CDI, *Clostridioides difficile* infection; IBD, inflammatory bowel disease; MR, Mendelian randomization; UC, ulcerative colitis.

**Fig 2:**
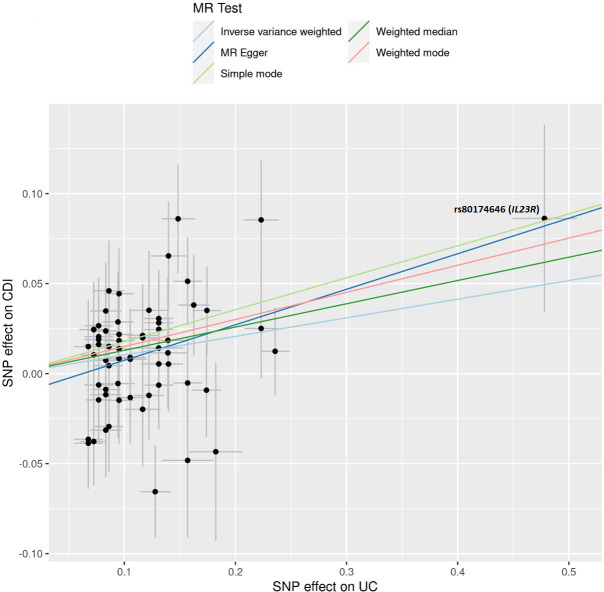
SNP effects on ulcerative colitis (UC) against SNP effects on *Clostridioides difficile* infection (CDI). Each black point represents an individual SNP. The SNP with the largest effect size on UC (β = −0.48) and CDI (−0.09) is rs80174646, which is an intronic variant in *IL23R*. Negative beta values for the exposure SNP are flipped to the positive direction to represent the effect allele, with corresponding flipping of the outcome beta value to reflect this change.

Sensitivity analyses were then performed to assess for causal effects by disease subtypes. In Crohn’s disease (CD), 142 SNPs were identified from Liu et al., and of these, 99 SNPs were found to be independent after clumping. Further, 97 were present in the outcome data set (CDI). There was no association between CD and CDI using MR Egger (β = 0.08, *P* = 0.19; OR 1.09 [95% CI 0.96–1.23]), but there was a significant association using IVW (β = 0.06, *P* = 0.008; OR 1.06 [95% CI 1.02–1.11]) (Table 2). There was no heterogeneity in either test (MR Egger [*P* = 0.49], IVW [*P* = 0.51]) or evidence of directional pleiotropy (*P* = 0.698). Additionally, the MR-PRESSO global test indicated no pleiotropy (*P* = 0.53). For ulcerative colitis (UC), there were 89 SNPs identified from Liu et al., and of these, 69 SNPs were found to be independent after clumping. Further, 62 were present in the outcome data set (CDI). There was a significant association between UC and CDI using MR Egger (β = 0.20, *P* = 0.01; OR 1.22 [95% CI 1.05–1.41]) and IVW (β = 0.10, *P* = 0.0003; OR 1.11 [95% CI 1.05–1.17]) (Table 2). There was no heterogeneity in either test (MR Egger [*P* = 0.48] or IVW [*P* = 0.45]) or evidence of directional pleiotropy (*P* = 0.18). Again, the MR-PRESSO global test indicated no pleiotropy (*P* = 0.44). Output from the scatter plot (Table S7) showing SNP effects on UC and CDI (Fig. 2) revealed an SNP, rs80174646-A, which was substantially protective for both UC (β = −0.48) and CDI (β = −0.086). This SNP annotates to an intronic region of the *IL23R* gene.

Reverse causality was also evaluated (i.e., genetically mediated CDI causal for IBD). Of the 154 SNPs, 7 remained independent after clumping and 5 were present in the outcome data set. MR Egger and IVW methods indicated no association with IBD (*P* = 0.67 and *P* = 0.42, respectively) (Table 2). There was no evidence of heterogeneity in the two tests (*P* = 0.88 and *P* = 0.94, respectively). There was no evidence of pleiotropy when evaluated using the Egger intercept (*P* = 0.76) or the MR-PRESSO global test (*P* = 0.94).

## DISCUSSION

There are several major findings from this paper. First, we describe a novel variant on chromosome 6, which is associated with susceptibility to CDI at genome-wide significance, reinforcing the concept of host immunity as an important contributor to CDI susceptibility. Second, results from the MR suggest that genetically predisposed UC is potentially causal for CDI, which may help explain the observed association between the two diseases. Third, the most notable SNP effect across diseases (UC and CDI), rs80174646-A, annotates to the *IL23R* gene, which may highlight an avenue for further therapeutic investigation.

There are two notable published GWASs for CDI with the first including 1,160 cases of CDI and 15,304 controls and the second including 988 cases of CDI and 13,632 controls (20, 21). Both studies highlight an association signal near chromosome 6. In the first study, rs114751021-A was linked to antibiotic-associated CDI in subset analyses (OR 2.42, 95% CI 1.84–3.11) (20). The rs114751021 variant annotates to the *SNORD117* gene on chromosome 6 and is near several *HLA* genes. In the second study, several variants on chromosome 6 were significantly associated with CDI at genome-wide significance (rs68148149-C, *P* = 8.06 × 10^−14^; rs3828840-T, *P* = 9.96 × 10^−14^; rs35882239-A, *P* = 8.18 × 10^−12^; rs71534541-C, *P* = 5.12 × 10^−11^; rs35222480-A, *P* = 9.88 × 10^−11^; rs116603449-T, *P* = 5.42 × 10^−10^), reinforcing the concept that genetic variation in this region contributes to CDI susceptibility. Importantly, the second study analyses were adjusted for age, body mass index, sex, ancestry, nursing home status, chemotherapy, diabetes, human immunodeficiency virus, transplant medications, corticosteroids, and antibiotic exposure, reducing confounding by co-morbidity and exposure. The lead variant in this work, rs68148149, is located between *HLA-DRB5* and *HLA-DRB6* and near *HLA-DRB1*. While the broad HLA association analyses did not reveal any statistically significant association, subset analysis of the HLA-DRB allele groups did suggest higher risk with the DRB1*15:01-DRB5*01:01 haplotype.

In our meta-analysis, we had a substantial gain in power with 3,500 cases of CDI, over three times what has been previously reported. A genome-wide significant variant, rs3134745-T, annotating to chromosome 6 (position 31242762), was associated with increased susceptibility to CDI. Using LDlink in a European ancestry population, the genome-wide variant (rs3134745) was not found to be in high LD with the lead variant from either of the previous studies: rs114751021 (*r*^2^ = 0.008) or rs68148149 (*r*^2^ = 0.025) (22). Thus, these variants may represent independent associations in the same region or highlight a common association with an unknown variant. Regardless, the reproducible signal on chromosome 6, near the MHC, strongly implicates host immunity in susceptibility to CDI.

We also identified a possible HLA allele group associated with CDI susceptibility, HLA-B*35:01. As described above, prior data highlight the DRB1*15:01-DRB5*01:01 risk haplotype in CDI susceptibility. In our data set, the DRB1*15:01 allele group had an association *P* value of 0.07. The DRB5*01:01 allele group was not available for testing, however. Ultimately, larger-powered studies will be beneficial to clarify causative HLA allele groups, given the small sample sizes of both studies. Interestingly, variation at the class II HLA genes has been found to be associated with rates of bezlotoxumab success implying that not only genetic variation at the MHC is important in disease susceptibility but also stratification of treatment response (23). The reason genetic variation at the MHC influences susceptibility to CDI remains unknown. However, as genetic variation at the MHC has been linked to susceptibility to several infections, aberrations in antigen recognition, processing, and presentation may represent a shared mechanism of disease pathogenesis (24–27).

There were also interesting genes that did not reach genome-wide significance but did reach a more nominal significance threshold. One example is rs11707141-A, which is an intergenic variant located between *RETNLB* and *TRAT1*. This variant is in high LD (*R*^2^ = 0.99) with a nonsynomous exonic variant (rs11708527: C59T/P20L) in *RETNLB*. (Supplementary Data, Table S8) *RETNLB* encodes the protein resistin-like beta, also known as RELM-Beta or FIZZ2. RELM-Beta mRNA has been shown to be expressed in goblet cells in the colon (28). RELM-Beta exerts local antimicrobial activity (29) in addition to contributing to the spatial segregation between epithelial cells and gut microbiota (30). RELM-Beta deficient mice infected with Citrobacter rodentium exhibit impaired CD4+ T-cell recruitment, reduced production of interleukin22 (IL-22), increased invasion of pathogens into colonic crypts, worsened inflammation, and higher mortality which reverses with RELM-Beta rescue via enema (31). Furthermore, RELM-Beta has been shown to exert species-specific antimicrobial effects, which can lead to loss of microbiome-mediated homeostasis and subsequent colitis (32). These data suggest that antimicrobial proteins secreted by goblet cells are integral to the host response in enteric infections. The potential genetic association between *RETNLB* and CDI as well as the functional work showing the role of RELM-Beta in protecting against enteric infections suggests that RELM-Beta should be investigated further in the pathogenesis of CDI and may represent a novel therapeutic target.

A second important finding is that genetic predisposition toward development of UC is potentially causal for CDI. IBD patients are more likely to be asymptomatic carriers of *C. difficile* implying the microbial dysbiosis or epithelial injury induced by IBD promotes a hospitable environment for this organism (3). However, there has been limited work investigating genetic links across these diseases. The advantage of using an MR approach to answer this question is that genetic variants are randomly distributed at conception and do not change over time regardless of environmental or medication exposures. Using MR, a potentially causal relationship was observed between genetically predisposed UC and CDI. It is worth noting that none of the IBD risk variants included in MR were found to be significant in the GWAS of CDI. This negative association may be due to modest effect sizes of individual variants that could not be picked up on this relatively small GWAS, or it may be that individual variants offer little direct influence on CDI susceptibility and polygenic risk is what drives the relationship. These results are important because they not only help clarify the directional relationship between IBD and CDI but they again implicate the importance of the host immune response in CDI as many of the IBD susceptibility variants are highly represented in immune pathways.

A final notable finding from these results is the effect of the variant rs80174646-A, annotated to the *IL-23R* gene, which was found to be protective in both UC and CDI in the MR. This variant is in high LD (*R*^2^ = 0.92) with a nonsynomous exonic variant (rs11708527: G1142A/R381Q) in *IL23R* (supplemental material, Table S8). An interesting study in 2013 showed that (i) human intestinal biopsies from patients with *C. difficile* colitis had increased staining of IL-23p19 in lamina propria cells compared to controls (1.33 ± 0.30 vs 0.7 ± 0.29, *P* = 0.008); (ii) mice lacking IL-23 signaling (IL-23p19^−/−^) had a significantly higher likelihood of survival than wild-type mice (100% vs 16.7%); and (iii) mice with IL-23 signaling neutralized by an anti-p19 antibody also exhibited improved survival (100% vs 50%) (33). These data suggest that blockade of IL-23 signaling is beneficial in CDI. However, there are conflicting data regarding IL-22 signaling and CDI. Because IL-23 is a potent inducer of IL-22, one would hypothesize based on the data above that reduction in IL-22 would be associated with a protective effect in CDI. However, several studies have shown the opposite, that IL-22 itself exerts a protective effect in CDI. Specifically, in mouse studies, IL-22 has been shown (i) to direct glycosylation of the gut microbiome, creating an unfavorable environment for *Clostrioides difficile*; (ii) reduce CDI-mediated colonic inflammation; (iii) limit the negative consequences of systemic dissemination of commensal bacteria through complement-activated bacterial phagocytosis; and (iv) improve morbidity and mortality associated with infection (34–36). Ultimately, the relationship between IL-23 signaling and CDI is likely complex and remains incompletely understood. With the increasing adoption of targeted IL-23 therapies in the treatment of IBD, it will be of benefit to investigate the infection rates of CDI across exposed and unexposed patients as well as recurrence rates and disease severity. Such epidemiologic studies may yield important insights into the therapeutic effect and therapeutic potential of this pathway in CDI.

There are some limitations of this work that are important to acknowledge. First, this meta-analysis of CDI was relatively small and thus may underestimate genetic contribution to CDI susceptibility. Second, individual-level genotypes were not available for all cohorts. Therefore, HLA association analysis may also underestimate association effects. The suggestive signal at HLA-B*35:01 should be tested for association in larger cohorts. Third, while MR results suggest a causal relationship between genetically predicted UC and CDI, results should be replicated in additional cohorts with individual-level data on confounders of interest (i.e., antibiotics and health care exposure). Fourth, the results of this paper were gathered from a European ancestry cohort. Therefore, these results may not be generalizable to other populations. Finally, these results do not shed light on CDI severity or recurrence which would be beneficial to investigate in follow up studies, as prior small cohort studies have identified possible genetic associations (23, 37, 38).

In summary, we report the largest GWAS of CDI to date reproducing the association between genetic variation on chromosome 6 (near the MHC) and susceptibility to CDI. We also provide data to support a causal relationship between genetically predicted UC and CDI. These results should prompt investigation into the mechanisms by which host immunity confers increased susceptibility to UC and CDI as such work could improve our understanding of the relationship between these two diseases and perhaps identify novel therapeutic targets for this important patient population.

## SUPPLEMENTAL MATERIAL

Supplemental Figures msphere.00567-24-s0001.docx Figures S1 to S16.

Supplemental Material msphere.00567-24-s0002.docx Supplemental text.

Supplemental Tables msphere.00567-24-s0003.xlsx Tables S1 to S7.

ASM does not own the copyrights to Supplemental Material that may be linked to, or accessed through, an article. The authors have granted ASM a non-exclusive, world-wide license to publish the Supplemental Material files. Please contact the corresponding author directly for reuse.
