# Supplementary material for: Genetic insight into the relationship between inflammatory bowel disease and Clostridioides difficile infection
Source: mSphere. 2024 Oct 22;9(11):e00567-24. doi: 10.1128/msphere.00567-24 (PMC11580397; doi:10.1128/msphere.00567-24)

**Supplementary Figures**

**Figure 1**. SNP effects on Inflammatory Bowel Disease (IBD) against the SNP effects on *Clostridioides difficile* infection (CDI). Each black point represents an individual SNP.


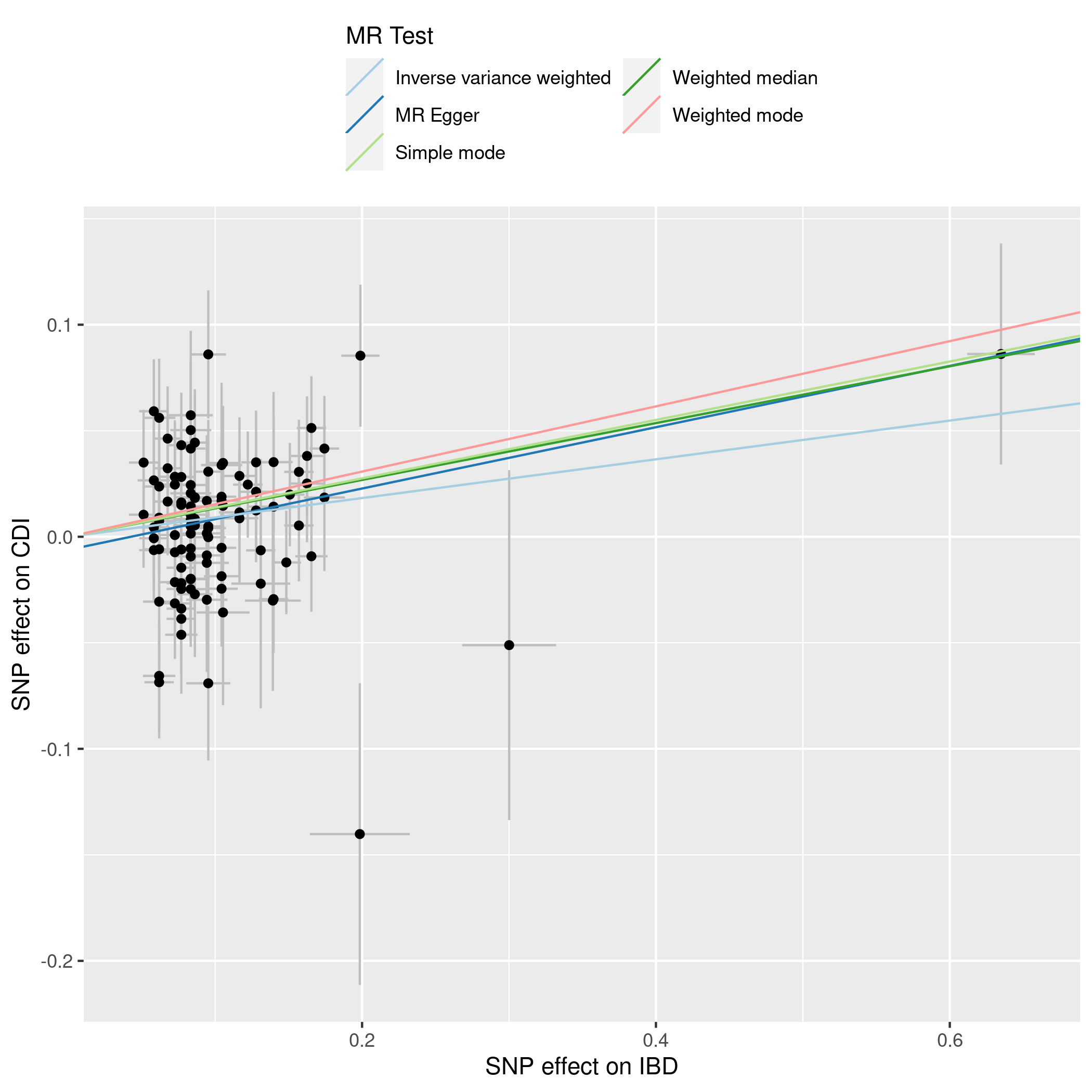


**Figure 2.** Relative Mendelian randomization (MR) estimates for each MR method (MR Egger, IVW) as well as single SNP tests for Inflammatory Bowel Disease ( IBD) on *Clostridioides difficile* infection (CDI).

**
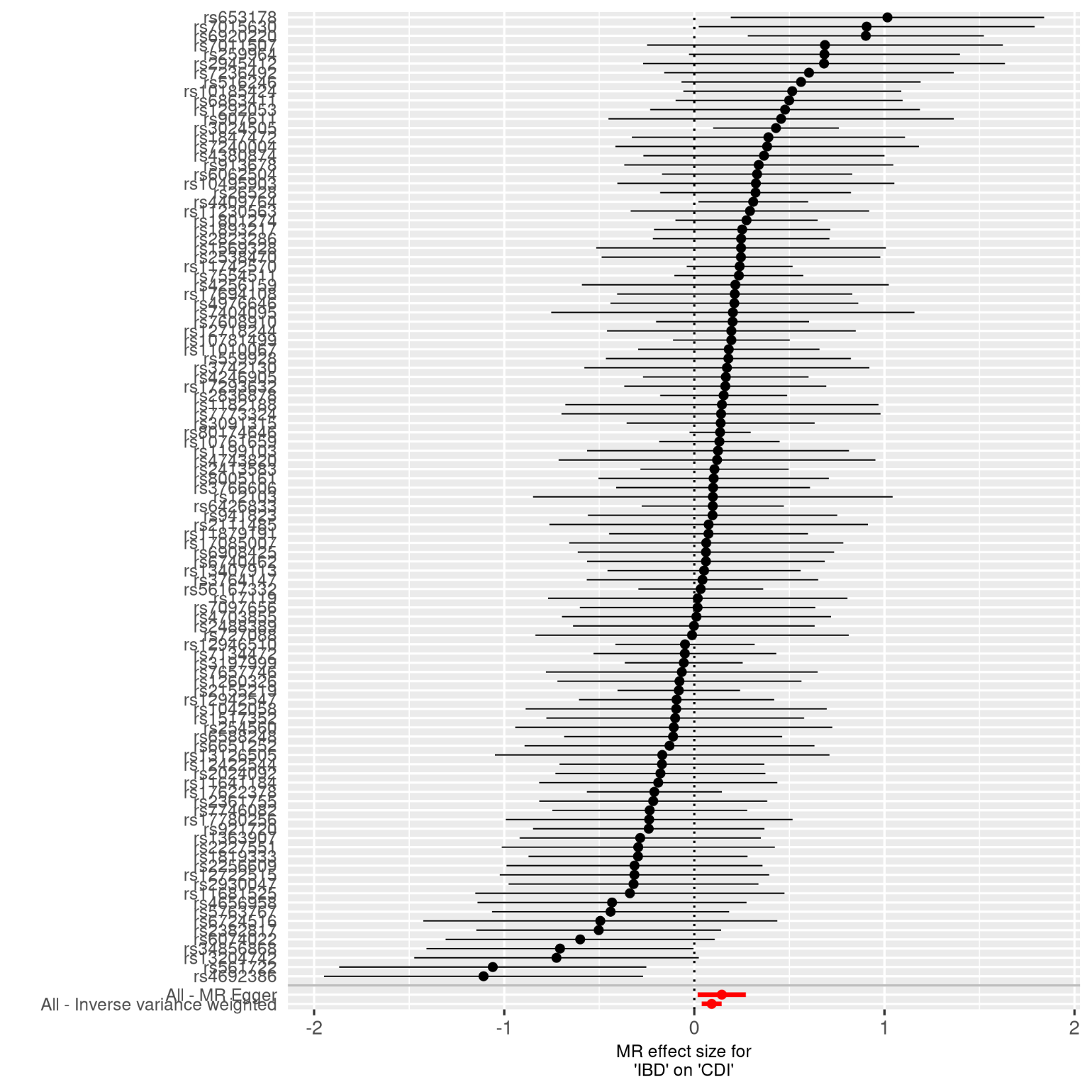
**

**Figure 3.** MR effects of IBD on CDI after leave-one out analysis. Each point represents the MR estimate after the corresponding SNP was excluded from analysis.

**
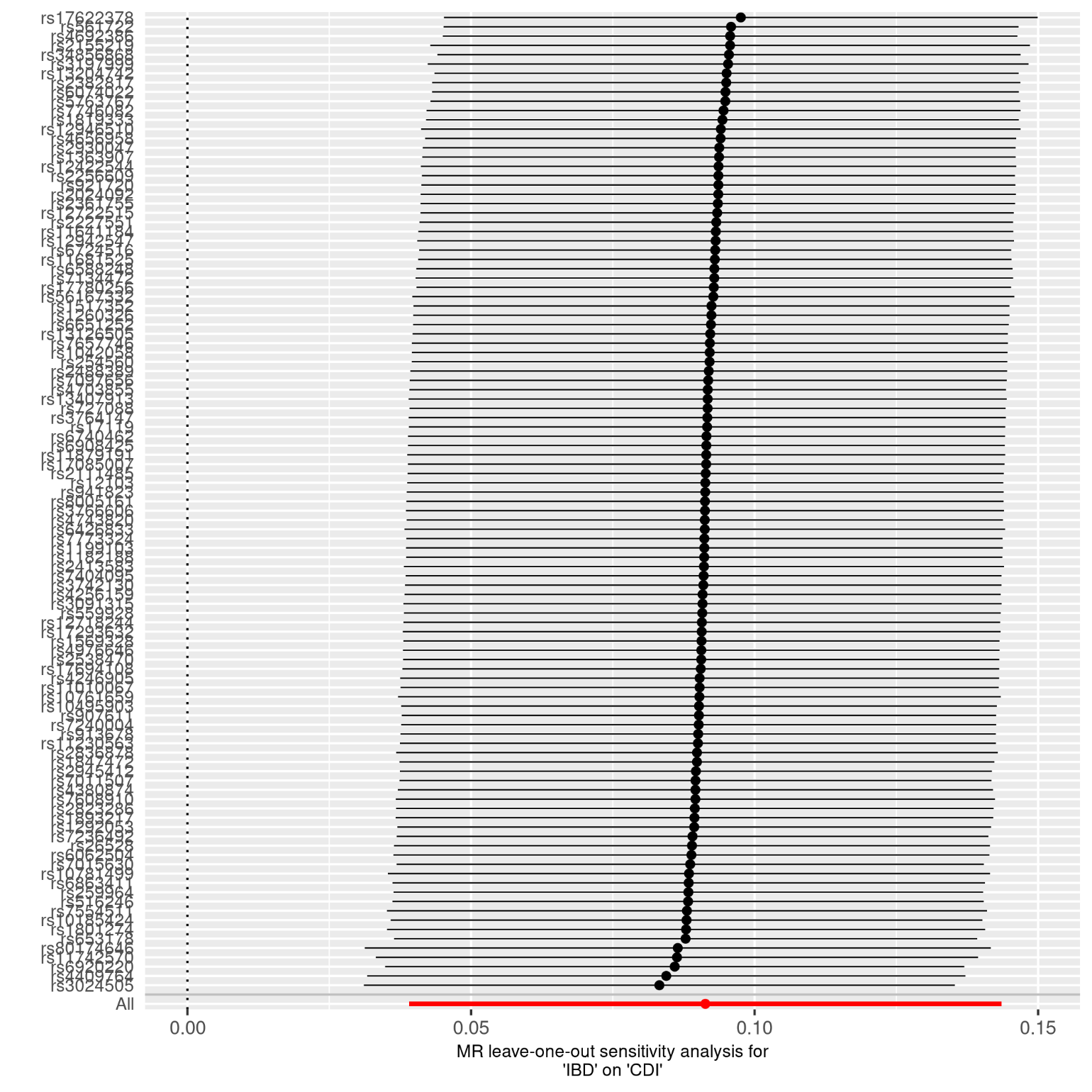
.**

**Figure 4.** Pooled estimates of each MR method for IBD on CDI. Each point represents a SNP with the x-axis representing the effect of the SNP and y-axis representing the inverse of the standard error.


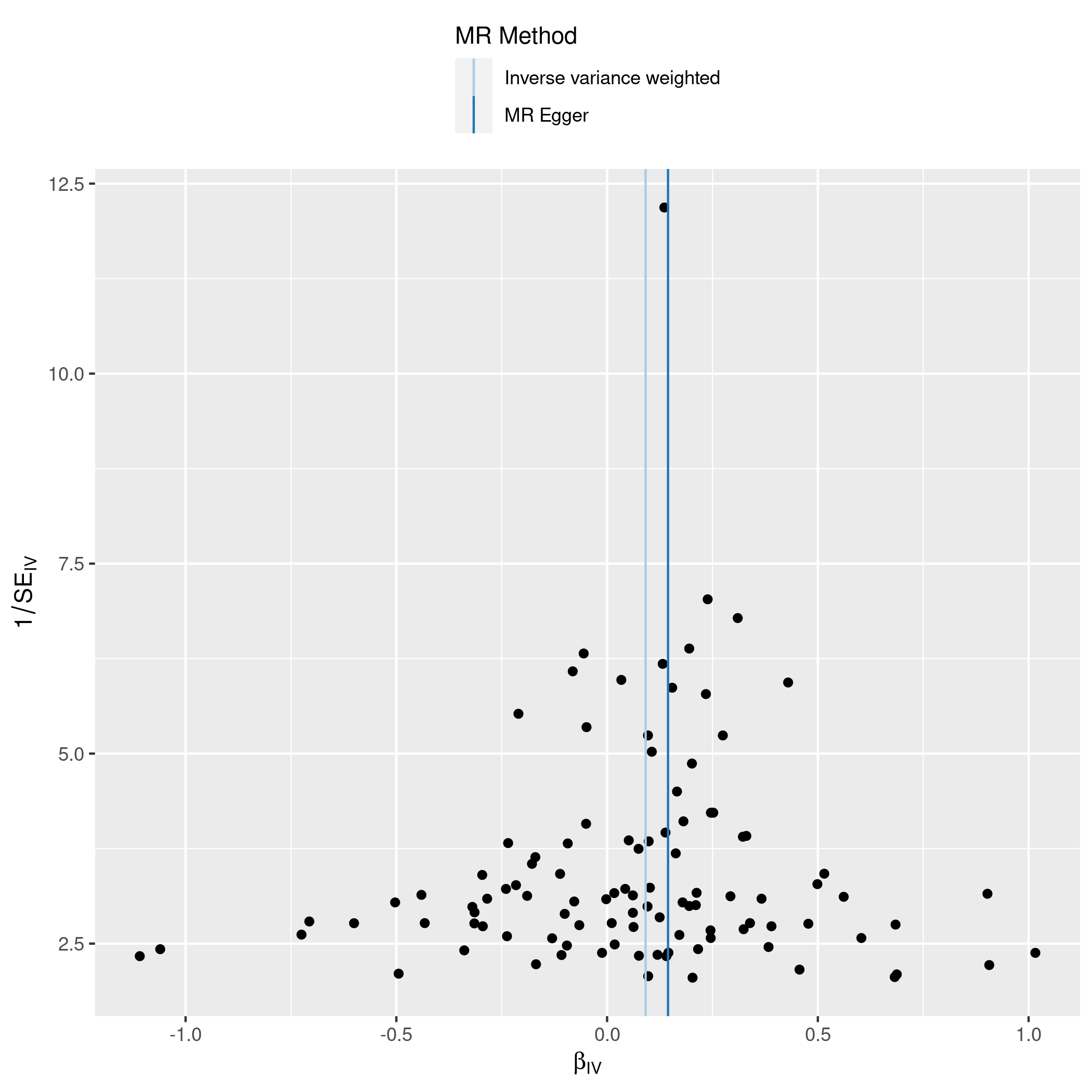


**Figure 5.** SNP effects on Crohn’s disease (CD) against the SNP effects on CDI. Each black point represents an individual SNP.


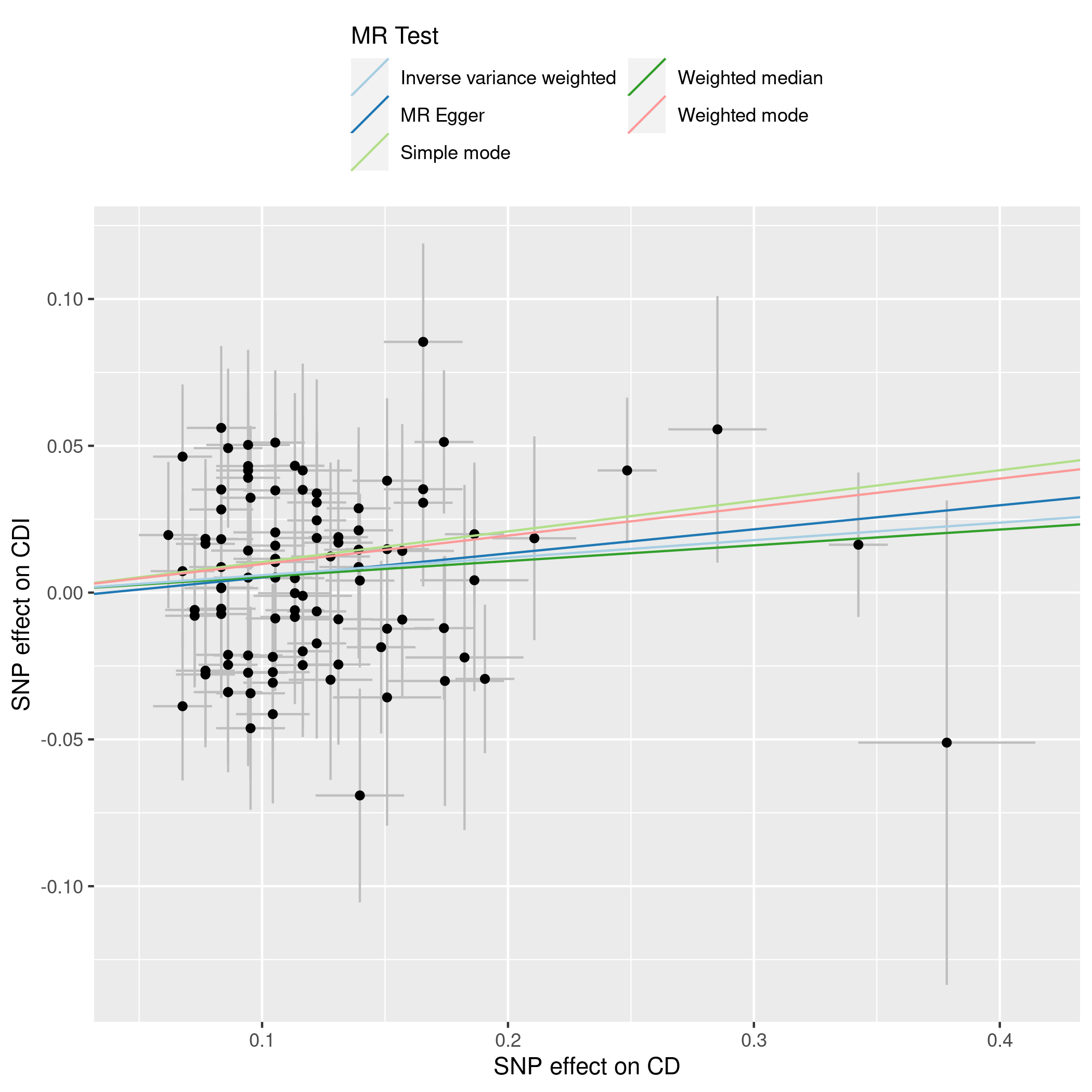


**Figure 6.** MR estimates for each MR method (MR Egger, IVW) as well as single SNP tests for Crohn’s disease (CD) on CDI


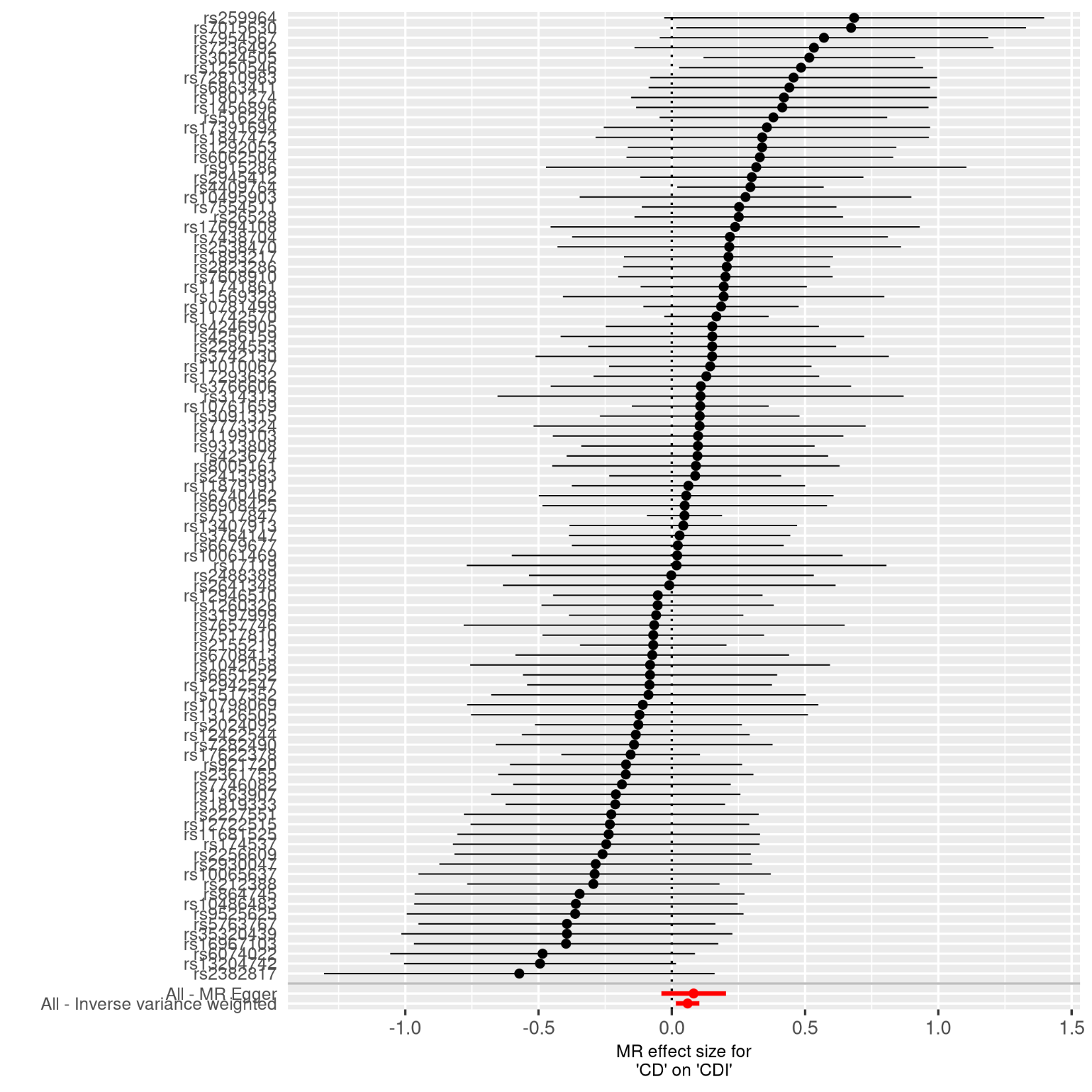


**Figure 7.** MR effects of Crohn’s disease (CD) on CDI after leave-one out analysis. Each point represents the MR estimate after the corresponding SNP was excluded from analysis.


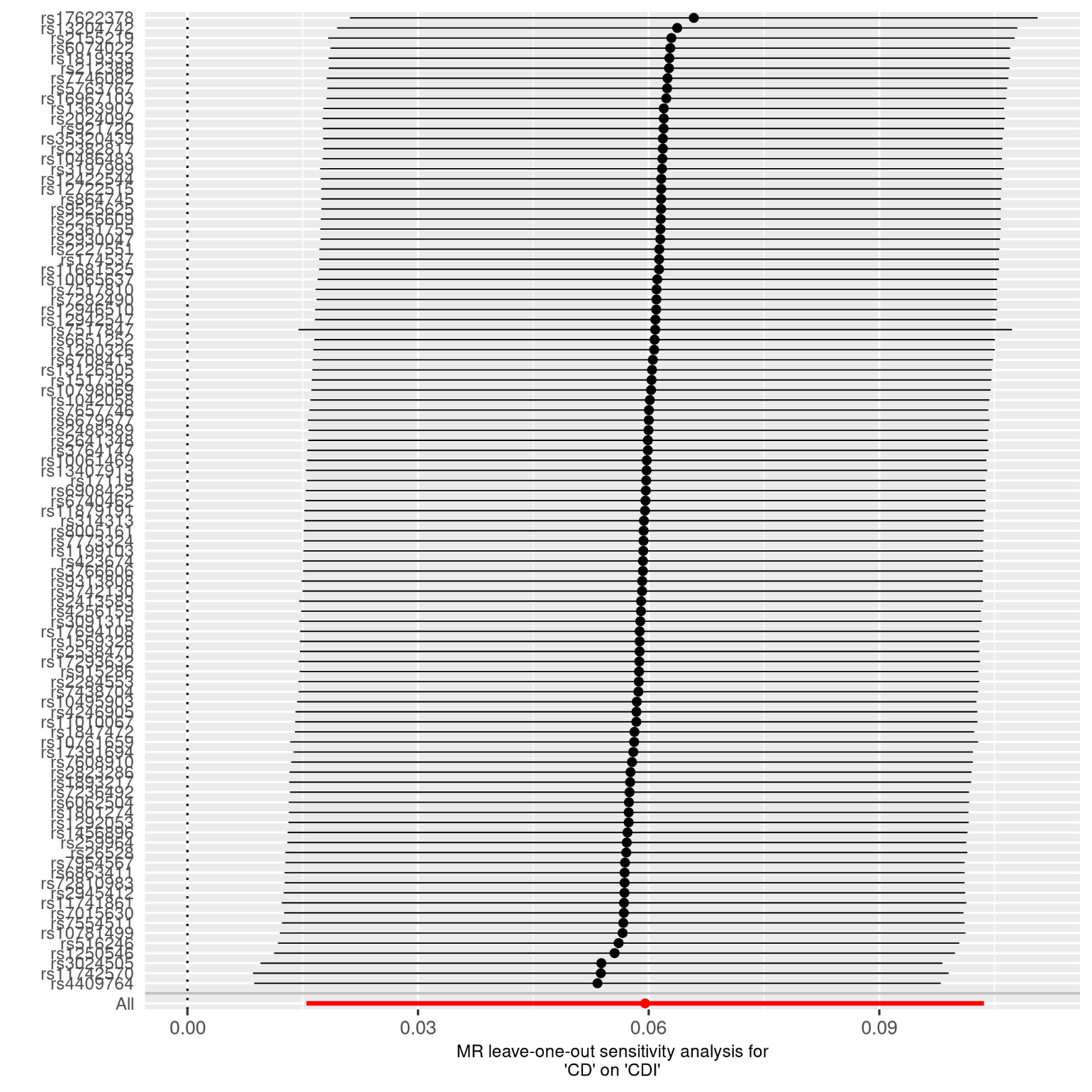


**Figure 8.** Pooled estimates of each MR method for Crohn’s disease (CD) on CDI. Each point represents a SNP with the x-axis representing the effect of the SNP and y-axis representing the inverse of the standard error.


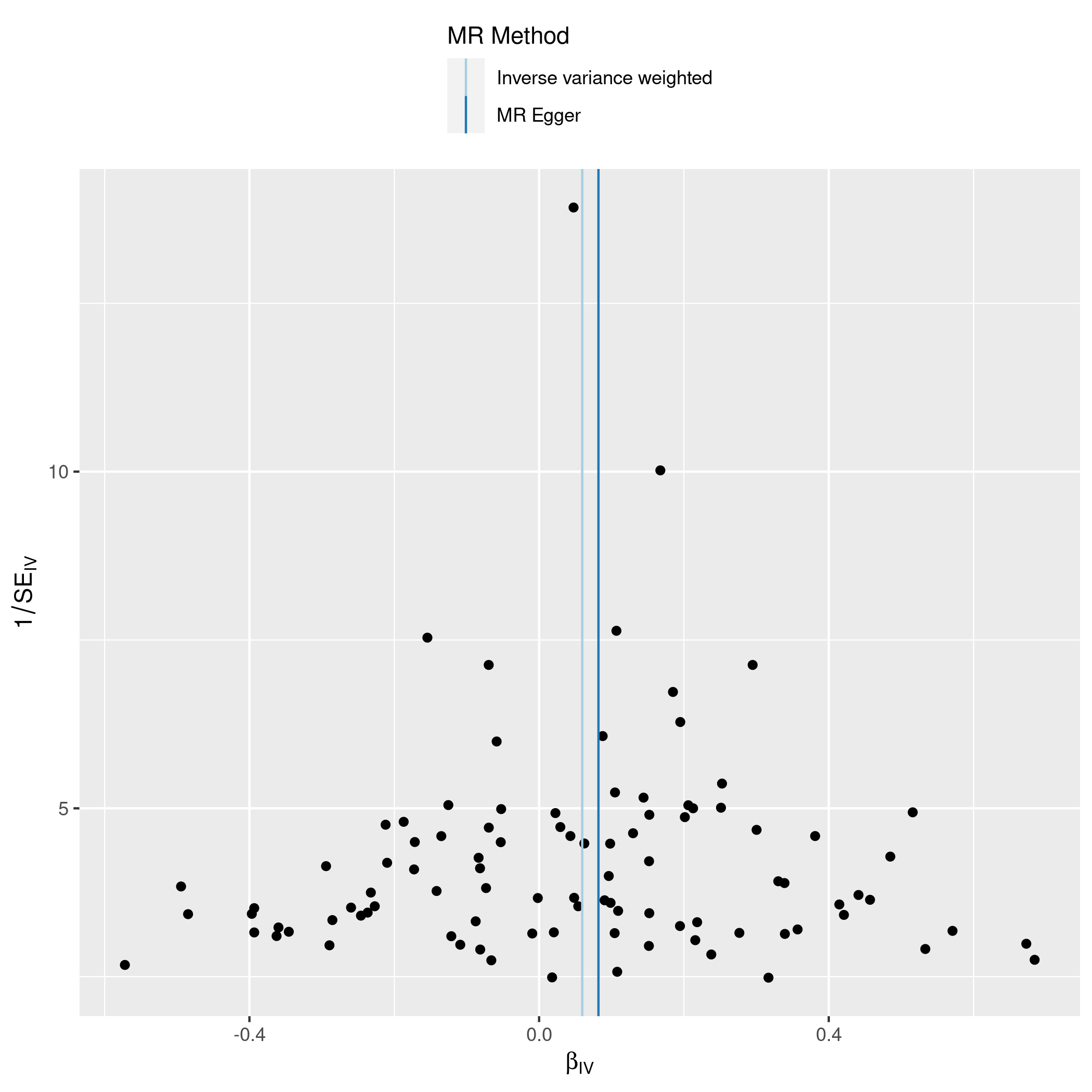


**Figure 9.** SNP effects on ulcerative colitis (UC) against the SNP effects on CDI. Each black point represents an individual SNP.


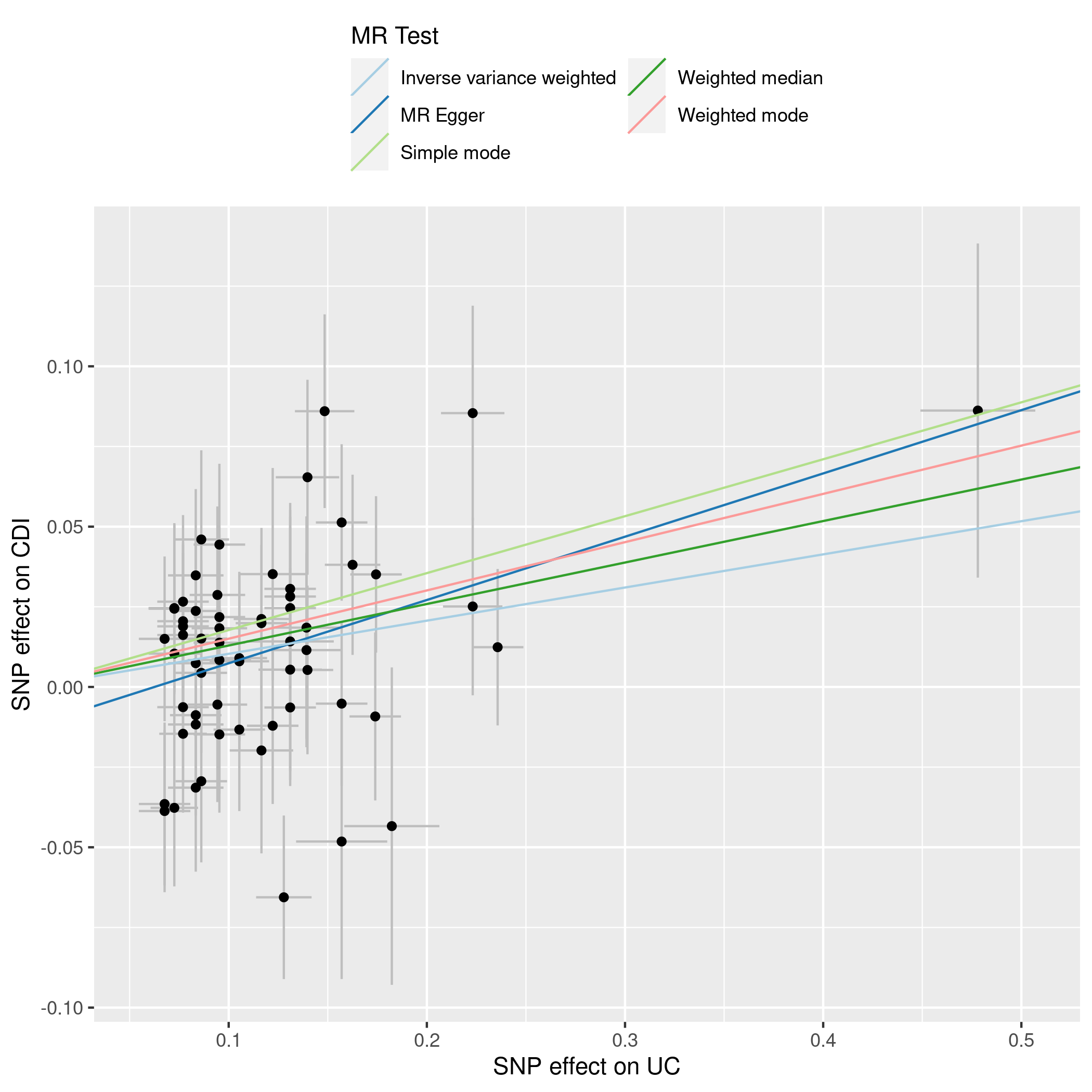


**Figure 10.** MR estimates for each MR method (MR Egger, IVW) as well as single SNP tests for ulcerative colitis (UC) on CDI


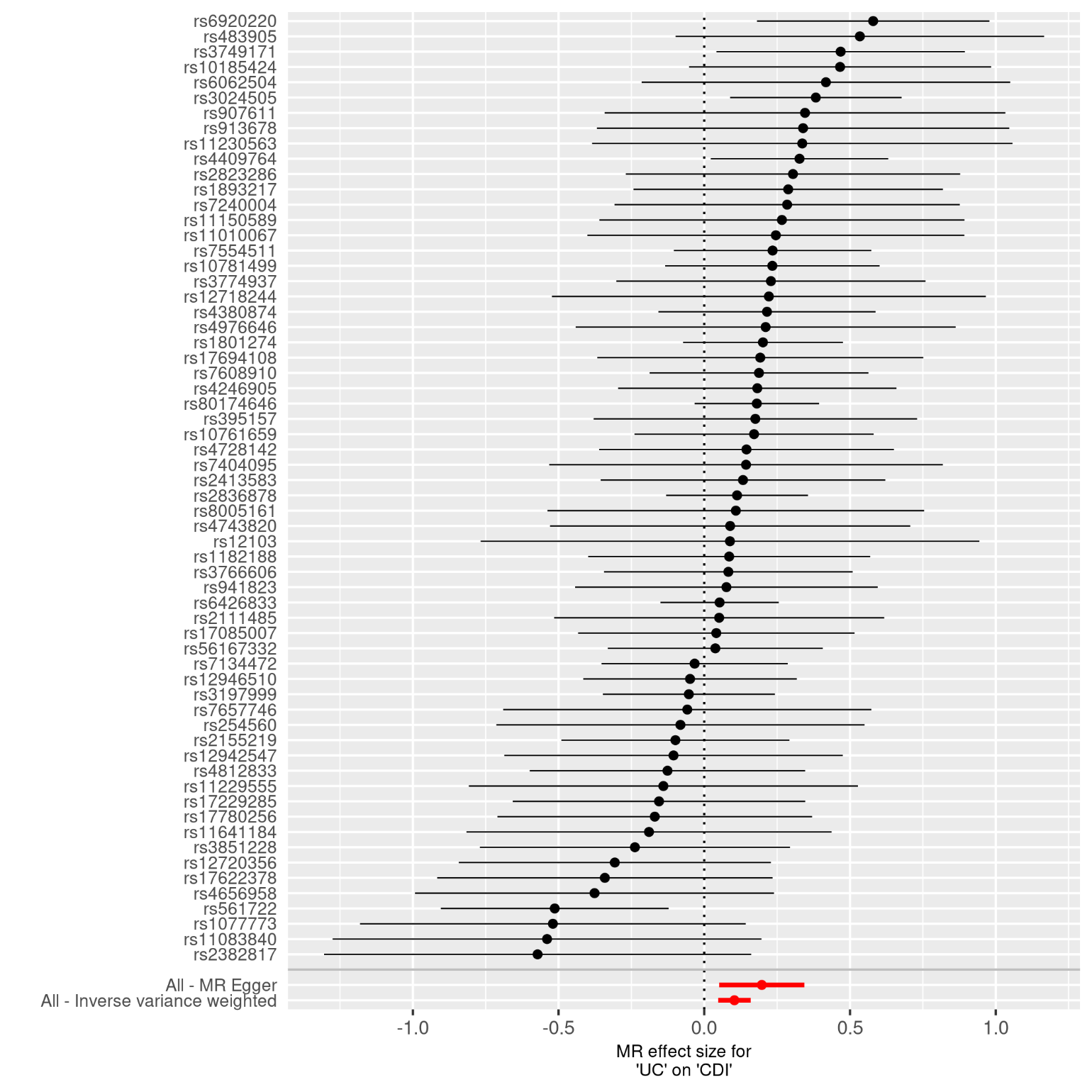


**Figure 11.** MR effects of ulcerative colitis (UC) on CDI after leave-one out analysis. Each point represents the MR estimate after the corresponding SNP was excluded from analysis.


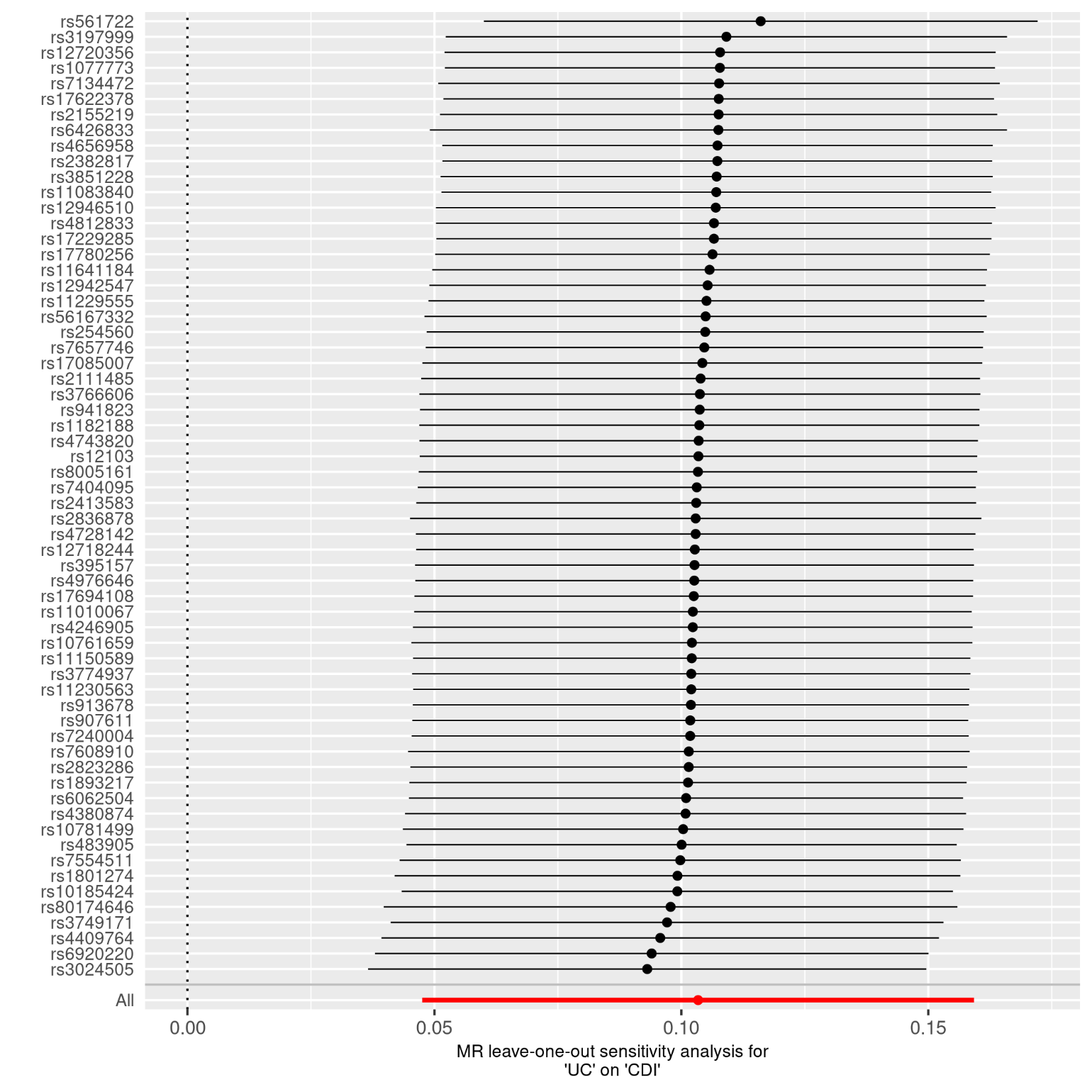


**Figure 12.** Pooled estimates of each MR method for ulcerative colitis (UC) on CDI. Each point represents a SNP with the x-axis representing the effect of the SNP and y-axis representing the inverse of the standard error.


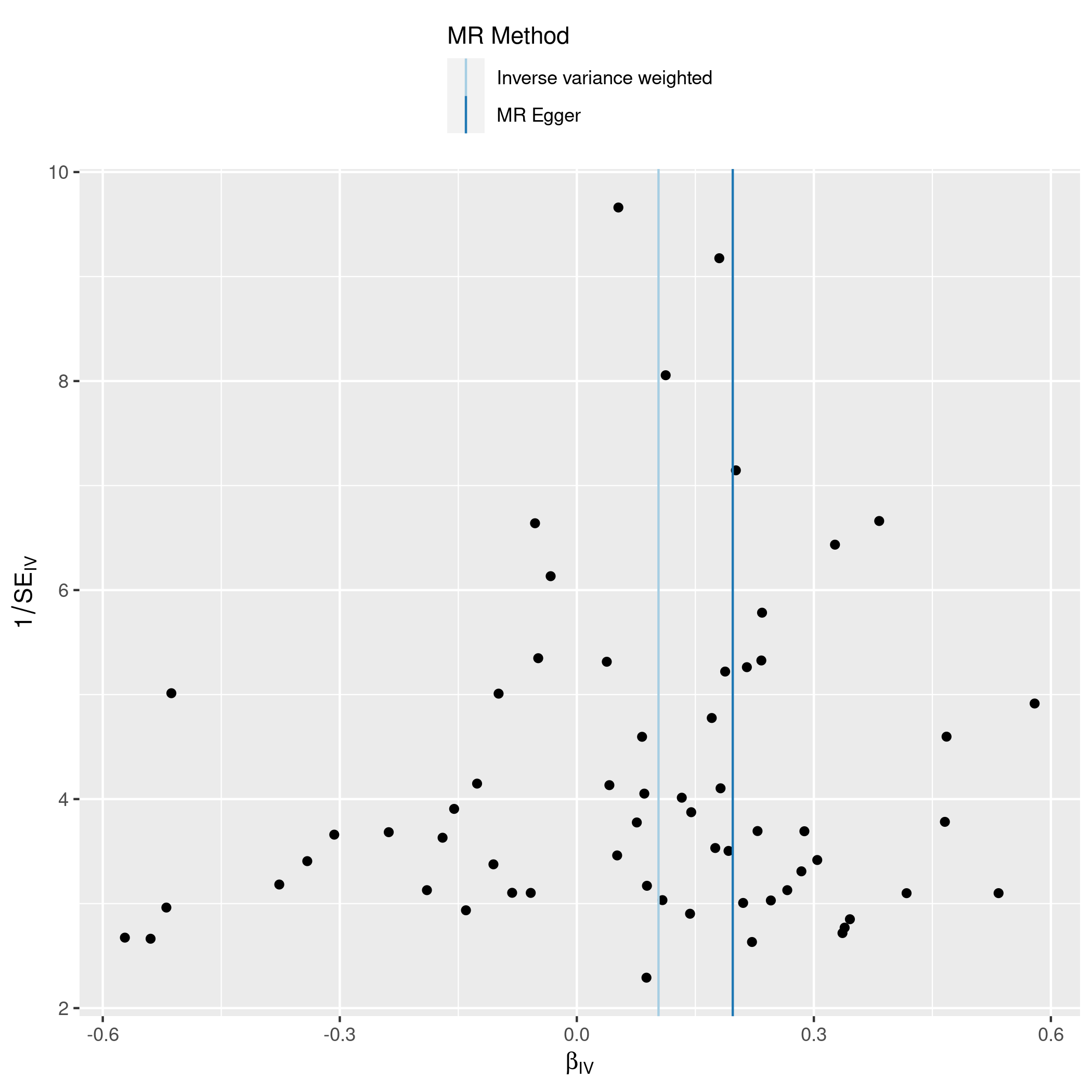


**Figure 13.** SNP effects on CDI against the SNP effects on IBD. Each black point represents an individual SNP.


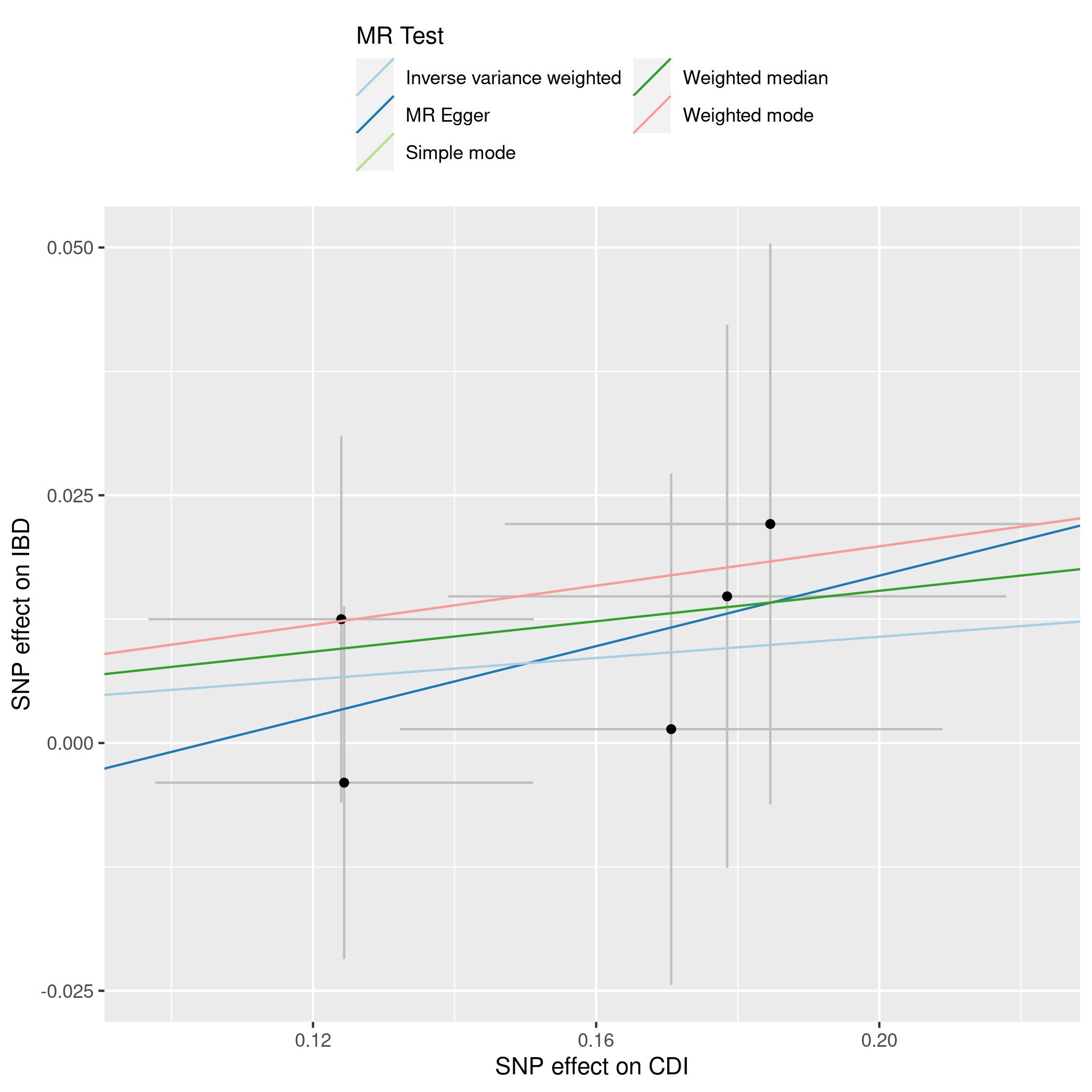


**Figure 14.** MR estimates for each MR method (MR Egger, IVW) as well as single SNP tests for CDI on IBD


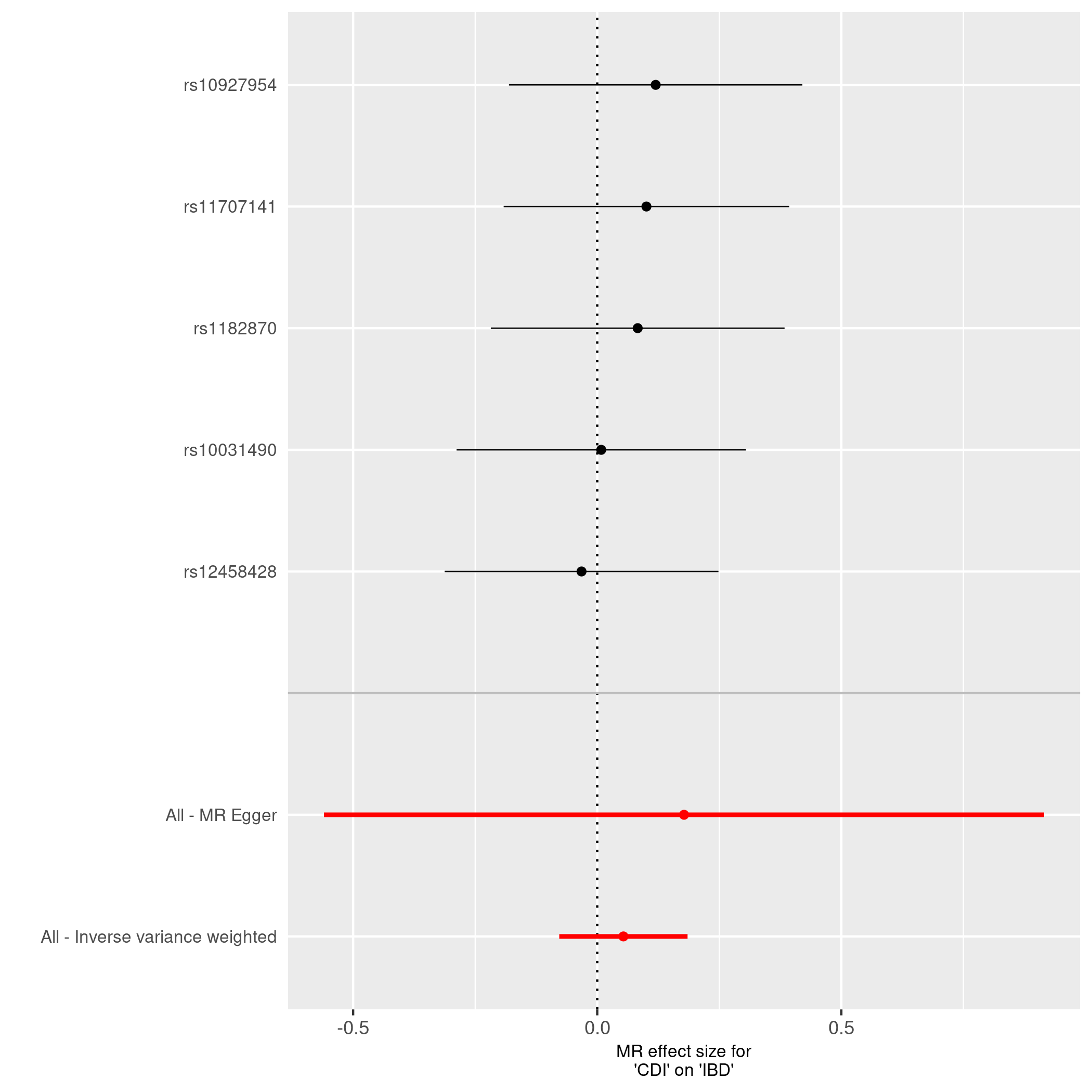


**Figure 15.** MR effects of CDI on IBD after leave-one out analysis. Each point represents the MR estimate after the corresponding SNP was excluded from analysis.


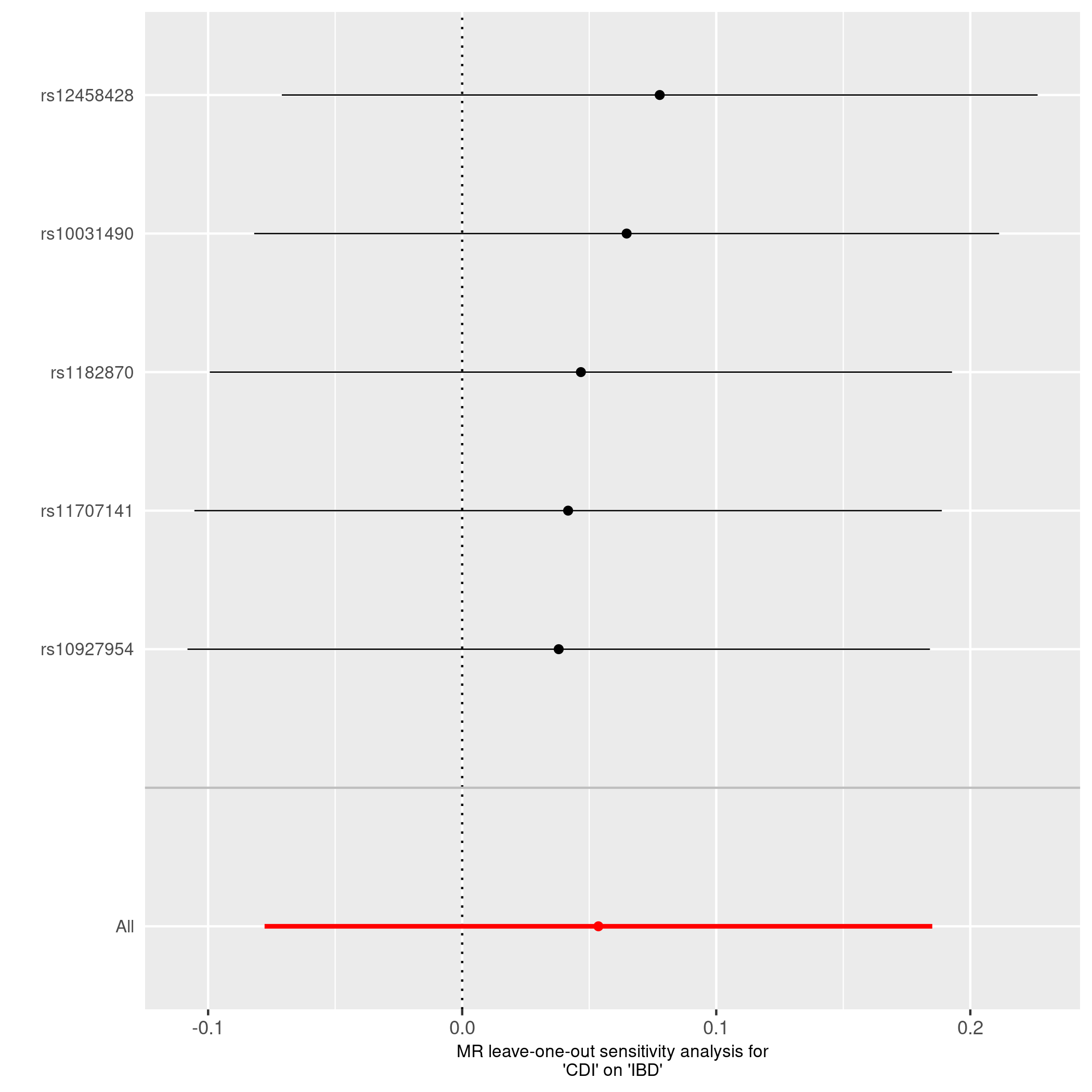


**Figure 16.** Pooled estimates of each MR method for CDI on IBD. Each point represents a SNP with the x-axis representing the effect of the SNP and y-axis representing the inverse of the standard error.


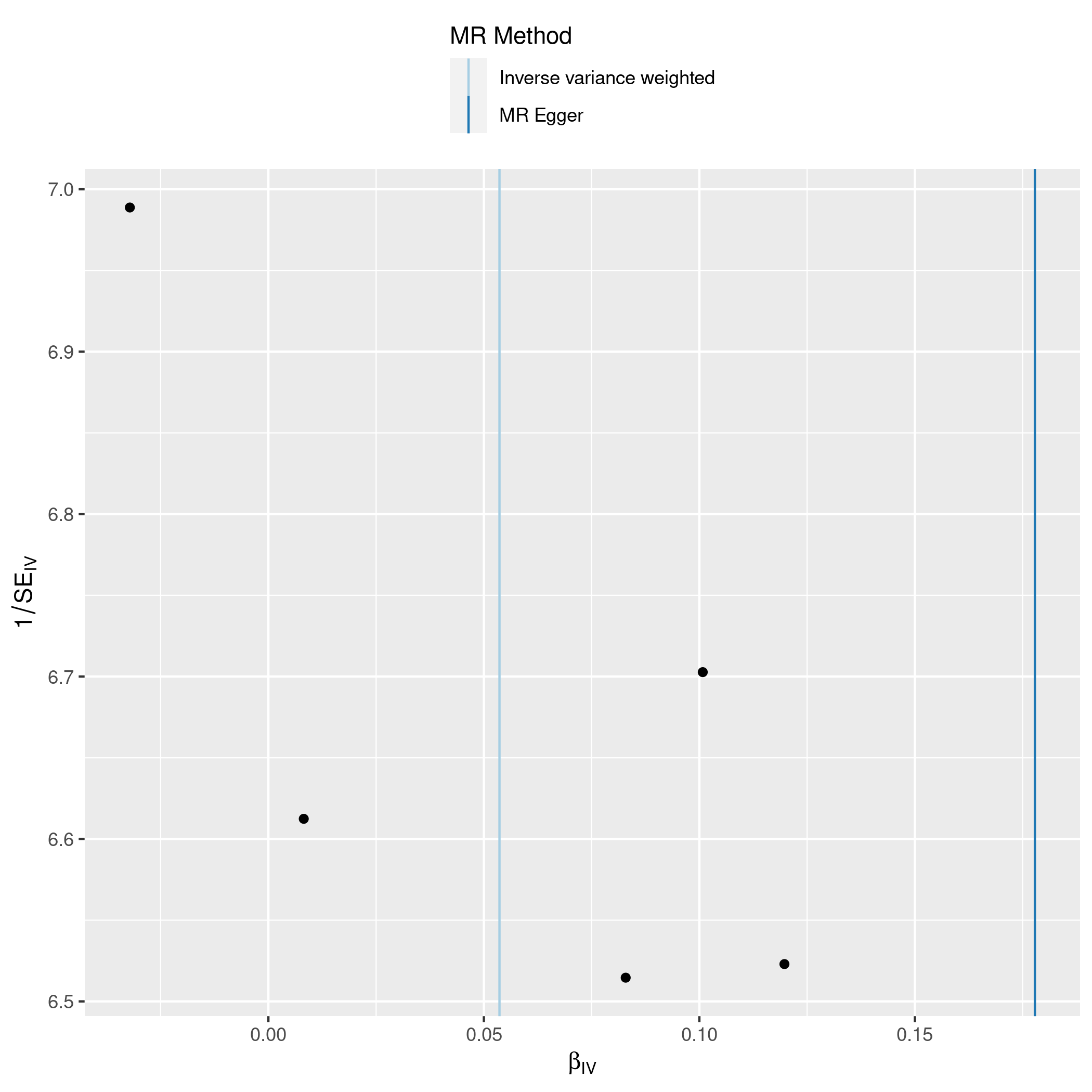

Supplement: Supplemental Figures — Figures S1 to S16. [file msphere.00567-24-s0001.docx]
