# Supplementary material for: Genetic insight into the relationship between inflammatory bowel disease and Clostridioides difficile infection
Source: mSphere. 2024 Oct 22;9(11):e00567-24. doi: 10.1128/msphere.00567-24 (PMC11580397; doi:10.1128/msphere.00567-24)
Supplement: Supplemental Material — Supplemental text. [file msphere.00567-24-s0002.docx]

**Table of Contents**

Data Cohorts, Genotyping, and Imputation 2

Genotype-Microbiome Analysis 4

Bi-Directional Assessment of Risk Variants Across CDI and IBD 5

High LD Nonsynonymous Variants 6

Data Availability Statement 7

References 8

**Data Cohorts, Genotyping, and Imputation**

FinnGen is a national biobank formed by a collaborative effort between the public and private sectors in Finland.^1^ In this cohort, clinical data from the public register is linked with individual-level genotype data and GWAS summary statistics are made publicly available. Details regarding genotyping, quality control, and imputation of release 5 can be found on the FinnGen website and associated publication.^2, 3^ Briefly, genotyping was performed using a custom Axiom FinnGen1 array and a non-custom genotyping array for a subset of participants. Investigators implemented strict quality control metrics accounting for sample-wise missingness rate, heterozygosity rates, relatedness, and sex discrepancies. Variants were excluded for call rates <98%, allele counts of less than 1, and deviation from Hardy-Weinberg Equilibrium (HWE). Imputation was performed using a population-specific SISu v3 imputation reference panel.

The United Kingdom Biobank (UKBB) is a population-based cohort of middle-aged individuals in the United Kingdom. Participants were between the ages of 40 and 69 and spanned a variety of socioeconomic and geographic backgrounds. Details of genotyping, quality control, and imputation can be found in the landmark UKBB manuscript.^4^ Briefly, genotyping was performed on the Affymetrix UK BiLEVE Axiom array or the Affymetrix UK Biobank Axiom® array, which had a high degree of overlap with over 95% commonality. Sample-wise and SNP-based quality control measures were implemented accounting for sample missingness, heterozygosity, and duplicates as well as SNP call rates, deviation from HWE, and batch effects. Imputation was performed using the IMPUTE4 program with several references’ panels including the Haplotype Reference Consortium and the merged UK10K/1000 Genomes phase 3 reference panels.

The Michigan Genomics Initiative (MGI) is a prospective institutional cohort of individuals who have received care at the University of Michigan Health System.^5^ Linkable electronic medical record and genotype data facilitate the study of precision health. For this study, the freeze 3 data release was used. Details of genotyping, quality control, and imputation can be found in the descriptive MGI manuscript.^5^ Briefly, genotyping data was performed using one of two customized versions of the Illumina Infinium CoreExome array. Quality control is described in the aforementioned manuscript, with exclusion based on study withdrawal, sex discrepancies, atypical gonosomal aberration, high kinship, low call rates, duplicates, and contamination. Furthermore, variant-wise quality control accounted for SNP call rates, deviation from HWE, and batch effects . Imputation was performed using Minimac4 (v1.0.0) with the reference panel Haplotype Reference Consortium.

All cohorts were restricted to European ancestry participants (**Supplementary Table 1**).

**Genotype-Microbiome Analysis**

Methods: Variants which associated with CDI in the conditional analysis were interrogated for association with microbial abundance. An *a priori* hypothesis that genotypes associated with a change in the abundance of Proteobacteria, Bacteriodetes, Firmicutes, and/or Actinobacteria was implemented based on available evidence^6^ and to limit penalties for multiple corrections. GWAS summary statistics for genotype-microbial abundance were extracted from the MiBioGen consortium analysis which included 18,340 individuals from 24 cohorts.^7^ Direction of effect is oriented to the CDI risk allele. Bonferroni corrected p-values were used to determine significance.

Results: The variants which associated with CDI were tested for association with microbial abundance of intestinal Proteobacteria, Bacteroidetes, Firmicutes, and/or Actinobacteria. No variants associated with abundance, in any phylum, at the Bonferroni corrected p-value of ≤0.0016. (**Supplementary Table 4**) One variant-phylum association (rs10031490-A/Actinobacteria) had a p-value of 0.02. Given this observation as well as data showing a reduction in the abundance of the genus Bifidobacterium (phylum: Actinobacteria)^6^, we subsequently evaluated the association between the rs10031490 variant and abundance of Bifidobacterium. The association was non-significant at p=0.067.

**Bi-Directional Assessment of Risk Variants Across CDI and IBD**

Methods: Chromosome and base position were used to identify any significant overlap across datasets (GRCh37/ hg19). First, genomic regions associated with CDI identified on COJO were tested for association with IBD. The IBD GWAS summary statistics were extracted for the genomic regions of interest from a publicly available European cohort meta-analysis of IBD susceptibility.^8^ Effect alleles were oriented towards the IBD risk increasing allele. A Bonferroni corrected p-value was considered significant. Next, genomic regions which associated with IBD were tested for association with CDI. Genome wide hits for IBD were identified in the meta-analysis described above.^8^ The CDI GWAS summary statistics from these hits were extracted from the CDI meta-analysis performed by the study team. A Bonferroni corrected p-value was considered significant.

Results: Of the 6 variants which associated with CDI, all were available in the IBD dataset. No variants associated with IBD at a Bonferroni corrected p-value. (**Supplementary Table 5**) Of the 159 variants which associated with IBD, all were available in the CDI dataset. No variants associated with CDI at a Bonferroni corrected p-value. (**Supplementary Table 6**)

**High LD Nonsynonymous Variants**

Methods: There were two variants identified in the data analyses that were of clinical and methodologic interest (rs80174646, rs11707141). The rs11707141 variant associated with CDI in the GWAS meta-analysis at a p<1e-05 and a nearby gene (*RETNLB*) was biologically plausible to influence disease pathogenesis. The rs80174646 variant is associated with IBD and had the strongest common effect across IBD and CDI in the Mendelian Randomization. As these variants were in non-exonic locations, we sought to identify if any nearby nonsynonymous variants were in high linkage disequilibrium (LD) with these index variants.

The primary GWAS data from publicly available IBD GWAS summary statistics^8^ referenced in this paper and the CDI meta-analysis conducted herein served as the data source for these analyses. Ancestry was restricted to European only. First, all independent SNPs were extracted from the GWAS dataset. Second, all variants within 500 kilobases upstream and downstream from the index SNP with a p-value for the trait < 10^-5^ were annotated. Genes were annotated by proximity (nearest gene to the index variant), presence of a high LD nonsynonymous variant in the index gene, and tissue expression in the ileum, sigmoid colon, or transverse colon [derived from GTEX data]. Third, all synonymous or non-coding variants were excluded from the dataset. Fourth, the LD was calculated between the index SNP and the remaining coding variants using R2. All coding variants with an R2 above 0.8 were included. Finally, the coding variant with the highest R2 was reported.

Data Availability Statement

FinnGen summary statistics are available at https://finngen.gitbook.io/documentation/v/r5/data-description#summary-association-statistics. UKBB individual-level data are available upon application ([https://ukbiobank.ac.uk](https://ukbiobank.ac.uk/)). MGI individual-level data are not publicly available due to privacy requirements. GWAS meta analysis summary statistics for CDI will be made available on the publicly available GWAS Catalog.

1. Kurki MI, Karjalainen J, Palta P, et al. FinnGen: Unique genetic insights from combining isolated population and national health register data. *medRxiv*. 2022:2022.03.03.22271360. doi:10.1101/2022.03.03.22271360

2. FinnGen. FinnGen Documentation of R5 release. 2021.

3. Kurki MI, Karjalainen J, Palta P, et al. FinnGen provides genetic insights from a well-phenotyped isolated population. *Nature*. 2023/01/01 2023;613(7944):508-518. doi:10.1038/s41586-022-05473-8

4. Bycroft C, Freeman C, Petkova D, et al. The UK Biobank resource with deep phenotyping and genomic data. *Nature*. 2018/10/01 2018;562(7726):203-209. doi:10.1038/s41586-018-0579-z

5. Zawistowski M, Fritsche LG, Pandit A, et al. The Michigan Genomics Initiative: A biobank linking genotypes and electronic clinical records in Michigan Medicine patients. *Cell Genom*. Feb 8 2023;3(2):100257. doi:10.1016/j.xgen.2023.100257

6. Martinez E, Taminiau B, Rodriguez C, Daube G. Gut Microbiota Composition Associated with Clostridioides difficile Colonization and Infection. *Pathogens*. Jul 8 2022;11(7)doi:10.3390/pathogens11070781

7. Kurilshikov A, Medina-Gomez C, Bacigalupe R, et al. Large-scale association analyses identify host factors influencing human gut microbiome composition. *Nature genetics*. Feb 2021;53(2):156-165. doi:10.1038/s41588-020-00763-1

8. Liu JZ, van Sommeren S, Huang H, et al. Association analyses identify 38 susceptibility loci for inflammatory bowel disease and highlight shared genetic risk across populations. *Nature genetics*. 2015/09/01 2015;47(9):979-986. doi:10.1038/ng.3359
